# Profile of analgesic prescription by medical specialties: A comparative study of symptomatic management in emergency care between orthopedics and other specialties–A retrospective observational brief report

**DOI:** 10.1371/journal.pone.0335629

**Published:** 2025-11-17

**Authors:** Luis Fernando Penna, Adhan A. Wu, Ricardo Madureira, Ricardo Luiz A. Fonseca, Renata Kobayasi Zelada, Fernando Ganem, Christian V. Morinaga

**Affiliations:** 1 Emergency Department, Hospital Sírio Libanês, São Paulo, Brazil; 2 Hematology Department, Hospital das Clinicas da Faculdade de Medicina da USP, São Paulo Brazil; 3 Orthopedic Emergency Department, Hospital Sírio Libanês, São Paulo, Brazil; 4 Hospital Sírio Libanês, São Paulo, Brazil; Galgotias University, INDIA

## Abstract

**Study objective:**

This study aims to analyze whether specific medical specialties are associated with potentially inappropriate analgesic prescriptions by examining and identifying variables related to both patient and prescriber profiles that influence certain analgesic choices.

**Methods:**

This is a retrospective longitudinal observational cohort study conducted based on medical records and charts from the Emergency Department (ED) at Hospital Sírio-Libanês (HSL), Bela Vista Unit, São Paulo, Brazil, from 2019 to 2022. It includes patients treated for pain with symptomatic medication administered alone or in combination, with outcomes assessing the efficiency of associations and the need for additional drugs.

**Results:**

Among 154,404 adult ED visits, 16,787 patients met the inclusion criteria. Orthopedics had a higher proportion of male patients (45.0%) with an older average age of 48.1 (±14.7) years. Additionally, 60.6% of these cases were classified as having a relatively urgent risk, and only 2.1% were emergencies. Orthopedic specialists had a higher likelihood of prescribing Level 2 and Level 3 analgesics, as well as being more likely to require rescue medication.

**Conclusion:**

The “Orthopedics” specialty prescribed more potent and a higher number of initial analgesics compared to other specialties, with a greater proportion of patients receiving rescue medications.

## Introduction

### Background

Pain is an extremely common symptom and one of the main reasons for seeking medical care in both the United States [1] and Worldwide [2,13–23]. Complaints such as low back pain [31] and headaches [3] rank among the most prevalent, particularly in Brazil, where EDs are commonly utilized for managing these symptoms.

Specialist intervention in pain management within ED settings remains insufficiently explored in the scientific literature. While numerous protocols and guidelines exist for pain management in EDs globally [12,29,30,31], the diversity and inconclusiveness of these studies complicate appropriate opioid use in ED environments [6,23], leading to highly variable and often belief-driven prescribing practices [6,8]. Specialists such as neurologists, geriatricians, physiatrists, rheumatologists, anesthesiologists, and orthopedists contribute predominantly in chronic pain contexts [14], focusing on prescribing extended-release opioids [10,13–15]. However, there is limited data regarding their role in managing acute pain in emergency scenarios [14].

Paradoxically, acute pain management predominantly occurs in EDs [14,29], where professionals often lack specific training for the pharmacological and multidisciplinary approach required [3,5–16]. This study analyzes the role of orthopedic specialists in managing acute pain in emergency settings, considering the technical scope of their expertise.

### Importance

This research seeks to enhance acute pain management in EDs, involving orthopedic specialists. It also addresses the lack of studies evaluating symptomatic prescription profiles across medical specialties in EDs.

### Goals of this investigation

This study aims to identify issues, biases, and sociocultural factors (from both patients and prescribers) influencing prescribing practices. Additionally, impacts stemming from these practices may alert healthcare professionals, fostering reflective and improved prescribing behaviors.

## Materials and methods

### Study design and setting

This is a retrospective longitudinal observational cohort study based on medical record reviews for primarily orthopedic complaints referred to orthopedic specialists in the ED of Tertiary Hospital, from 2019 to 2022.

### Selection of participants

Participants included patients who visited the ED and received symptomatic treatment for pain. Inclusion criteria comprised adults (≥18 years), initial analgesic prescription in the ED, pain levels ≥6 on the World Health Organization (WHO) pain scale, and treatment occurring between 2019 and 2022. Pain scores were standardized using the Numeric Rating Scale, ranging from 0 (no pain) to 10 (worst possible pain), to allow objective measurement of pain, a uniform comparison of pain levels across patients and management outcomes. Exclusion criteria were incomplete digital records (missing anamnesis or vital signs) to ensure integrity and accuracy of the data analyzed. Study variables included demographics (gender, age, marital status, education), triage data (risk classification, vital signs, pain score), and prescription data (attack medications, rescue medications, drug step per WHO scale, and prescriber specialty).

### Methods of measurement

The study was conducted in an emergency department with a well-established pain management protocol. According to this protocol, pain is reassessed one hour after the administration of the initial analgesic. If the patient’s reported pain level at that time remains above their individual comfort threshold, a second dose or alternative analgesic is prescribed. The choice of analgesic is made at the discretion of the attending physician, based on clinical judgment and patient-specific factors. Although the protocol recommends reassessment at one hour, in practice, this interval could be shorter in cases of evident pain escalation or prolonged in situations influenced by service dynamics, such as high patient flow or the patient’s temporary unavailability due to diagnostic procedures. For the purposes of this study, the need for additional analgesic prescriptions was considered an indicator of initial pain control failure.

Quantitative data are expressed as means and standard deviations, while qualitative data are represented as counts and percentages. Epidemiological, triage, and prescription variables were compared between orthopedics and other specialties using unpaired Mann-Whitney U test for continuous variables and Chi-square test for categorical variables.

Logistic regression models with 95% confidence intervals were used to evaluate the association between orthopedic classification and two outcomes: prescription of WHO level 3 analgesics (opioids) and prescription of three or more analgesic medications. For each outcome, two models were constructed: (1) an unadjusted model and (2) a multivariable model adjusted for age, sex, hospitalization status, triage risk classification, and heart rate. The selection of covariates for adjustment was based on clinical plausibility and their potential role as confounders. Age and sex are well-established factors that influence both pain perception and response to analgesic treatment. Hospitalization status was included as a proxy for clinical severity and may reflect more intense or prolonged pain. Triage risk classification identifies patients with higher acuity, who may require more aggressive analgesia. Finally, heart rate was included as a physiological marker of stress or pain, which could influence clinical decisions regarding the choice and intensity of analgesic prescriptions.

Statistical significance was set at p < 0.05. We conducted analyses using R software version 4.2.2.

## Results

During the study period, there were 154,404 emergency department visits by adult patients, of which 16,955 met the inclusion criteria and 168 (1.0%) had incomplete medical records and were excluded. The resulting sample of 16,787 visits are summarized in Table 1.

**Table 1:** Epidemiological data of the study sample (n = 16.787).

	Total (n = 16,787)	Orthopedics (n = 3,832)	Others (n = 12,955)	
**Average age (years ± SD)**	45.0 (±15.1)	48.1 (±14.7)	44 (±15.1)	< 0.01
**Masculine gender**	6,259 (37.3%)	1,723 (45.0%)	4,536 (35.1%)	< 0.01
**Marital status**				< 0.01
** Married**	9,366 (55,8%)	2,331 (60,8%)	7,035 (54.3%)	
** Single**	5,894 (35,1%)	1,109 (29,0%)	4,785 (36.9%)	
** Others**	1,527 (9.1%)	392 (10,2%)	1,135 (8.8%)	
**Educational level**				< 0.01
** Higher education**	13,480 (80.3%)	3,203 (83.5%)	10,277 (79.3%)	
** Complete high**	2,204 (13.1%)	390 (10.2%)	1,814 (14.0%)	
** Uninformed or up to basic level**	1.103 (6.6%)	239 (6.2%)	864 (6.7%)	
** Hospitalization**	1.438 (8.6%)	69 (1.8%)	1,369 (10.6%)	< 0.01
**Triage classification**				< 0.01
** Red and Orange**	355 (2.1%)	26 (0.7%)	329 (2.5%)	
** Yellow**	5,765 (34.4%)	1,182 (30.8%)	4,583 (35.4%)	
** Green and Blue**	10,175 (60.6%)	2,567 (67.0%)	7,608 (58.7%)	
** Unclassified**	492 (2.9%)	57 (1.5%)	435 (3.4%)	
**Average blood pressure (mmHg ± SD)**	97.6 (±13.4)	98.1 (±12.3)	97.4 (±13.7)	< 0.01
**Heart rate (bpm)**	84 (±15.6)	79.5 (±13.1)	85.3 (±16.1)	< 0.01
**Scale of pain**	8.2 (±1.1)	8.1 (±1.1)	8.2 (±1.0)	0.04

The epidemiological profile of the study population showed a majority of women (62.6%), with an average age of 45.0 (±15.1) years, mostly married (55.8%), and with a higher level of education (80.3% with a university degree). In the group treated by Orthopedics, there was a higher proportion of men (45.0%) and an older average age of 48.1 (±14.7) years compared to the ‘Other’ group (including all other specialties except Orthopedics).

Regarding the patient profile, 60.6% were classified as green and blue level of urgency, and only 2.1% were red and orange emergency cases. The risk classification was even lower among Orthopedics patients, with only 0.7% classified as red and orange emergency cases and 30.8% as yellow urgent.

In terms of pain management, the average pain score was 8.2 (±1.1), with similar scores between the Orthopedics group and other specialties (8.1 ± 1.0 vs 8.2 ± 1.1).

Regarding clinical condition, patients showed stable hemodynamic profiles, with an average mean arterial pressure (MAP) of 97.6 (±13.4) mmHg and a heart rate of 84 (±15.6) bm. Orthopedic patients tended to have lower heart rates compared to those in other specialties, with an average of 79.5 (±13.1) bm.

The most commonly used analgesics, both in the initial treatment (Table 2a) and in rescue treatment (Table 2b), are summarized below.

**Table 2:** (a) Attack medication used. (b) Recue medication used.

(a) Attack medication used			
ATTACK MEDICATION			
Medication		Proportion by Category	
**Dipyrone**	9199	**Weak analgesics**	32.15%
**ketaprofen**	5308	**NSAIDs**	25.00%
**Scopolamine**	4010	**Corticosteroids**	15.35%
**Dexamethasone**	3600	**Others (includes scopolamine)**	14.24%
**Tramadol**	3130	**Opioids**	13.18%
**Other NSAIDs**	2268		
**Other corticosteroids**	1043		
**Other opioids**	855		
**Weak analgesics**	521		
**Others**	296		
**Total**	**30230**		
**(b)** **Recue medication used**			
**RESCUE MEDICATION**			
**Medication**		**Proportion by Category**	
**Tramadol**	804	**Opioides**	41.92%
**Morphine**	648	**Corticosteroids**	17.97%
**Dipyrone**	472	**Weak Analgesic**	16.28%
**Dexamethasone**	382	**NSADs**	15.54%
**Ketaprofen**	338	**Others**	8.26%
**Others**	300		
**Other corticosteroids**	270		
**Other NSADs**	226		
**Weak Analgesics**	119		
**Other opioids**	69		
**Total**	**3628**		

The most used attack analgesics were dipyrone (9,199), followed by ketoprofen (5,308) and scopolamine (4,010). More than half of the attack medication fell into either the category of weak analgesics (32.15%) or NSAIDs (Non-Steroidal Anti-Inflammatory Drugs) (25.00%). On the other hand, in terms of rescue medication profile, there was limited prescription of weak analgesics (16.28%) and NSAIDs (15.54%). A predominance of opioid use (41.92%) was observed, particularly tramadol (804) and morphine (648). In accordance with the WHO Analgesic Ladder, the aforementioned medications were classified according to their respective levels. The prescription pattern for pain management and the outcome regarding rescue medication requirements are summarized in Table 3.

**Table 3:** Pain prescription patterns and rescue medication outcomes.

	Orthopedics (n = 3.832)	Others (n = 12.955)	
**Maximum WHO level of attack medications**			<0.01
** Level 1**	1,601 (41.8%)	11,276 (87.1%)	
** Level 2**	1,975 (51.5%)	1,282 (9.9%)	
** Level 3**	256 (6.7%)	397 (3,0%)	
**Number of attack medications**			<0.01
** 1 drug**	785 (20.5%)	3,291 (25.4%)	
** 2 drugs**	1,158 (30.2%)	4,159 (32.1%)	
** 3 or more drugs**	1,889 (49.3%)	5,505 (42.5%)	
**Rescue medication administration**	636 (16.6%)	1.913 (14.8%)	0.006
**Time to first rescue (hours)**	2.6 (± 1.8)	2.7 (± 2.0)	0.41

Regarding the levels recommended by the WHO, there was a difference in the use of mild analgesics (level 1) as attack medication, with Orthopedics prescribing less frequently than other specialties (41.8% vs. 87.1%; p < 0.01).

The need for rescue medication was greater between orthopedists and other specialties (16.6% vs 14.8%), and the number of hours until the first rescue was similar between the two groups (2.6 hours).

**Fig 1:**
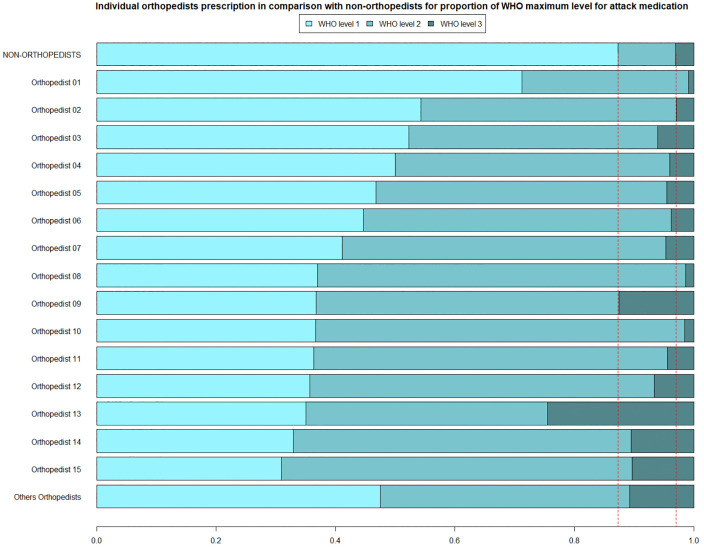
Individual orthopedists’ prescription in comparison with non-orthopedists for proportion of WHO maximum level for attack medication.

In Fig 1, we present the 15 orthopedists with the highest prescription volume in the study, corresponding to all on-call orthopedists in the department. Other orthopedists include members of the clinical staff who may occasionally issue prescriptions but whose overall volume is significantly lower than that of the on-call physicians.

The graph shows that, among all orthopedists studied, the proportion of prescriptions containing WHO Level 1 medications as the maximum potency is lower compared to non-orthopedists, suggesting a group-wide prescribing behavior. Regarding the proportion of prescriptions at WHO Level 3 of analgesia, only three orthopedists prescribe proportionally fewer of these medications than the non-orthopedist group.

Among Orthopedics patients, 20.5% received only one analgesic drug, 30.2% received two, and 49.3% received three or more. In contrast, among the Others group, 25.4% received a single analgesic drug, 32.1% received two, and 42.5% received three or more (p < 0.01).

**Fig 2:**
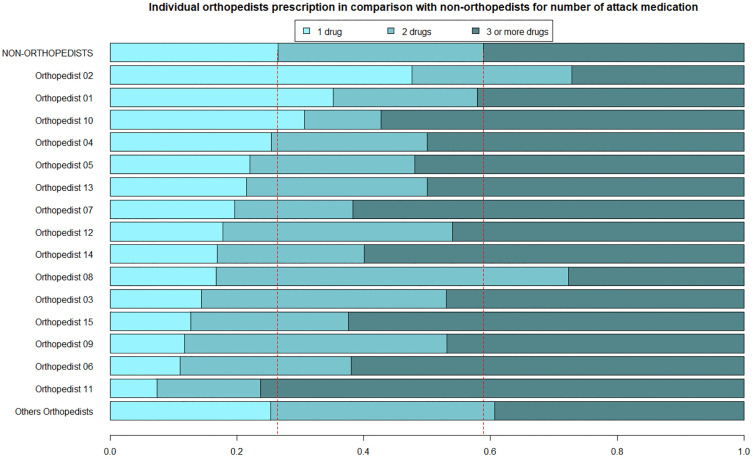
Individual orthopedists’ prescription in comparison with non-orthopedists for number of attack medication.

In Fig 2, with respect to the prescribing patterns of the 15 on-call orthopedists, the prescription of only one analgesic medication in the acute phase is more frequent in just 3 (20%) of these physicians. Similarly, the majority of orthopedists (n = 13, 86.7%) proportionally prescribe three or more analgesic drugs in the acute phase compared to the non-orthopedist group.

In Table 4, the unadjusted analysis, orthopedic classification was significantly associated with both outcomes. Patients classified as orthopedic had more than twice the odds of receiving a WHO level 3 analgesic (opioid) compared to non-orthopedic patients (OR: 2.26; 95% CI: 1.92–2.66; p < 0.01). Similarly, the odds of receiving three or more analgesic medications were also significantly higher among orthopedic patients (OR: 1.31; 95% CI: 1.22–1.41; p < 0.01).

**Table 4:** Odds ratios (OR) and 95% confidence intervals (CI) for the association between orthopedic classification and two outcomes: prescription of WHO level 3 analgesics (opioids) and prescription of three or more analgesic medications. Estimates are presented for unadjusted and multivariable models adjusted for age, sex, hospitalization status, triage risk, and heart rate.

ORTHOPEDIC PATIENTS				p-value
Model	OR for opioid use [95% CI]	p-value	OR for > 2 drugs	
**Unadjusted model**	2.26 [1.92–2.66]	<0.01	1.31[1.22–1.41]	< 0.01
**Adjusted model**	**3.40 [2.84–4.07]**	< 0.01	**1.38 [1.28–1.49]**	**< 0.01**

After adjusting for age, sex, hospitalization status, triage risk, and heart rate, both associations remained significant. The adjusted odds of opioid prescription among orthopedic patients increased to 3.40 (95% CI: 2.84–4.07; p < 0.01), and the adjusted odds of receiving three or more analgesics was 1.38 (95% CI: 1.28–1.49; p < 0.01). These findings suggest that orthopedic classification is an independent predictor of both stronger and more complex analgesic prescribing patterns in the emergency department setting.

## Limitations

This study has several limitations. First, the project was conducted at a single private healthcare center. As a result, although the findings align with studies from other regions regarding medical specialty and analgesic prescription patterns, the generalizability of these results may be limited due to the lack of a population-representative sample.

The second limitation is that medical specialty may serve as a proxy for the type of clinical condition treated. Different medical conditions may require different levels of analgesic prescription. Although we have adjusted the logistic regression model for hospitalization and risk classification to control for condition severity, future studies should incorporate more detailed diagnostic information to better isolate the independent effect of prescriber specialty.

The third limitation is the inability to determine how many patients presenting with acute pain also had underlying chronic pain. Although all included patients sought care for acute pain, the presence of chronic pain conditions was not systematically documented. Furthermore, data on patient comorbidities were not available. The absence of this information limits a more comprehensive understanding of pain intensity, treatment response, and analgesic needs, as both chronic pain and comorbidities—such as depression, anxiety, or previous opioid use—may significantly influence pain perception and the effectiveness of pain management strategies.

Another limitation is the scarcity of studies on this topic. Consequently, international sources, primarily from the United States and Canada, were utilized, posing challenges in adapting the findings to the sociocultural context, as opioid regulation policies and cultural approaches vary significantly.

Lastly, the absence of a more detailed analysis of the prescribers (e.g., training duration and location) limited the ability to gain a deeper understanding of the underlying reasons for prescription differences.

## Discussion

In this study, we chose to compare the characteristics of the analgesic prescription between orthopedists and other specialties. The main finding of our study demonstrated that the orthopedic specialty adopts a distinct approach to analgesic treatment management when compared to other specialties. Our results show that orthopedists prescribe more intensive analgesic treatments, with higher odds of utilizing WHO Level 2 and Level 3 analgesics, as well as combination therapy approaches.

The intensified analgesia observed in Orthopedics has also been reported in other studies comparing opioid prescription rates among medical specialties, indicating that specialties such as Physiatry, Pain Medicine, and Orthopedics are more frequently associated with higher opioid prescription rates [5,16,17]. A likely explanation for this is that these professionals are more exposed to patients with acute pain, which may be exacerbated by or compounded with chronic conditions. Additionally, these specialties often adjust analgesic prescriptions for these patients [16].

International comparisons should be interpreted with caution, as prescribing practices are shaped by a complex interplay of cultural, regulatory, and systemic factors. When compared to orthopedic physicians in India, those in our service had a different prescribing pattern for analgesics. Physicians in the Indian study prescribed an average of 1.46 analgesics per patient, predominantly NSAIDs [21], whereas in our study, the average was 2.6 analgesics per prescription, with weak opioids predominating. This highlights the variability in analgesic prescription rates within the same specialty, depending on the geographic location. This difference may be attributed to regional public policies. Some Asian countries have implemented more restrictive measures regarding the prescription and administration of opioids to prevent misuse, resulting in lower daily doses of opioids compared to Western countries [22]. However, we cannot infer that stricter opioid access policies necessarily reduce or increase the use of other analgesics, as some medications, such as NSAIDs, are over-the-counter and easily accessible. This could affect study outcomes, leading to varying results across different healthcare contexts. [23–24].

As noted by Sokoloff C [19], rapid analgesic delivery is associated with shorter emergency department stays. Both their study and ours focused on patients with WHO pain scores >6, in line with Canadian and Australian guidelines recommending initial analgesia within 25–30 minutes of arrival [25–26]. However, these recommendations refer to initial, not rescue, analgesia. Furthermore, patient satisfaction is more strongly linked to physician engagement than to pain outcomes [20]. Therefore, expedited rescue medication, without adequate reassessment, may not improve satisfaction and could contribute to suboptimal pain control, increased iatrogenic risk, and unnecessary costs [25–26].

Considering that the prescriber is primarily responsible for providing analgesics to patients [13], studies on their influence on analgesia have not always yielded expected results. In our study, for instance, despite the increased prescription of stronger analgesic medications, orthopedic patients had higher likelihood of requiring rescue medication compared to non-orthopedic patients. In the study by Barnett et al. [4], there was also no difference in patient revisits to emergency departments when comparing high-intensity and low-intensity prescribers, further indicating that greater quantities of analgesics do not necessarily result in better pain control.

The fact that emergency departments serve as the primary entry point for patients with painful conditions makes them prone to excessive opioid prescribing [27]. Multiple factors, such as overcrowding [18], the use of pain scales or guidelines [28] (pertaining to the emergency department itself), variability in professional training [15] and specialty [5,16,17] (pertaining to the prescriber), and individual patient characteristics [11], may coexist, highlighting the complexity of prescribing practices. However, good prescribing practices, aligned with major analgesia guidelines, have the potential to reduce chronic opioid use and dependence [29] and improve patient care.

Despite all arguments and the establishment of possible relationships cited in various previous studies, it is essential to consider that explanations for variations in prescribing patterns are not always found [13]. Often, they are based on the prescriber’s personal beliefs [3], contrasting with evidence-based clinical practice. This underscores a subjective aspect that is challenging to analyze scientifically.

In conclusion, opioid prescribing in the emergency department represents a multifactorial and context-dependent process, shaped by patient characteristics, institutional workflows, and physician clinical judgment. This study identified a distinct prescribing pattern among patients presenting with orthopedic complaints who were managed by orthopedic specialists. These findings highlight the need for more in-depth investigations to elucidate the determinants of opioid use in this setting and to support the development of targeted, evidence-based strategies for optimizing analgesic prescribing practices.
